# Aggression and Psychological Distress in Male and Female Anabolic-Androgenic Steroid Users: A Multigroup Latent Class Analysis

**DOI:** 10.3389/fpsyt.2021.629428

**Published:** 2021-06-04

**Authors:** Razieh Chegeni, Guy Notelaers, Ståle Pallesen, Dominic Sagoe

**Affiliations:** ^1^Department of Psychosocial Science, University of Bergen, Bergen, Norway; ^2^Optentia, The Vaal Triangle Campus of the North-West University, Vanderbijlpark, South Africa

**Keywords:** aggression, anabolic-androgenic steroids, multigroup latent class analysis, sex, psychological distress

## Abstract

The relationship between anabolic-androgenic steroid (AAS) use and aggression and psychological distress requires further elucidation. No previous study has examined whether the latent patterns of aggression and psychological distress are the same in male and female AAS users. Multigroup latent class analysis (MLCA) can be used to classify individuals into groups based on their responses on a set of variables, and to investigate measurement invariance across subgroups. We therefore conducted a MLCA to identify discrete subgroups of aggression and psychological distress in AAS users, and used measurement invariance to examine whether the identified subgroups can be applied to both sexes. We also examined the relationship between sex and subgroup belongingness. The sample comprised 206 AAS users (females = 58.30%) aged 14–56 (mean = 26.86, *SD* = 7.12) years. They completed questionnaires assessing demographics, AAS use, aggression, and psychological distress. Based on the MLCA, five subgroups were identified: high aggression moderate distress users (HAMoD: 07.63%), moderate aggression distress users (18.64%), moderate direct aggression-mild indirect aggression moderate distress users (22.95%), mild direct aggression-moderate indirect aggression-distress users (11.71%), and low aggression mild distress users (LAMiD: 39.06%). Although a homogenous five-class solution was the best model for both sexes, sex was significantly associated with the probability of subgroup membership. In particular, members of the HAMoD subgroup were more likely to be male whereas members of the LAMiD subgroup were more likely to be female. Our study provides novel empirical evidence of the idiosyncratic patterns of aggression and psychological distress among male and female AAS users.

## Introduction

Anabolic-androgenic steroids (AAS) comprise testosterone and its synthetic derivatives. Due to muscle mass building and strength enhancing properties AAS are typically used by elite athletes and bodybuilders. The global lifetime prevalence of AAS use (1) is estimated at 3.3% with a higher prevalence among males (6.4%) compared to females (1.6%).

Despite experiencing aesthetic and performance benefits (2, 3), users often report adverse physiological and psychological effects (4–8), such as lack of impulse control, hostility, and high levels of aggressive and violent behaviors (9–11). Indeed, a recent meta-analysis of 12 randomized controlled trials has shown that among healthy males, AAS administration increases self-reported aggression (12). However, considering the inconsistency of results across studies (13–22) it is not clear whether there are unobserved patterns of aggression and psychological distress among AAS users.

Investigating subgroups of AAS users can facilitate the identification of individuals and groups most sensitive to the negative psychological consequences of AAS use, as well as the development of targeted interventions (23, 24). Previous studies on subgroups of substance users have mainly focused on alcohol and tobacco use (25, 26). Still, few studies identifying subgroups of AAS and appearance- and performance-enhancing drug (APED) users have been conducted (27–30). However, no previous study has examined subgroups of AAS users based on their aggression and psychological distress profile, despite these factors being strongly associated with AAS use (5, 12, 31, 32). A multigroup latent class analysis [MLCA; (33)] is superior to traditional categorization of subgroups using cut-off points (34). Hence, we conducted a MLCA which is a powerful tool for classifying latent patterns of an observed variable as well as examining group differences simultaneously (33–35). We used the MLCA to identify subgroups of AAS users and gender differences in identified subgroups based on physical aggression, verbal aggression, anger, and hostility and psychological distress patterns.

Moreover, AAS use is predominantly a male practice and AAS use among females is an understudied area (1, 36). Relatedly, the few studies that have examined the side effects of AAS use among females are based on small samples (37–41). In addition, males and females tend to use different doses and types of AAS and believe that the doses and the types they choose is best fitting to their sex (37, 42). More importantly, females are an important group in clinical practice and policy making on AAS use. Thus, information about patterns of aggression and psychological distress in AAS-using females is important. Accordingly, we included female AAS users in the current study to identify their patterns of aggression and psychological distress and to examine subgroups of female and male AAS users. We also investigated whether there are unobserved sex differences in the identified patterns.

## Materials and Methods

### Participants

Participants comprised 206 current AAS-using bodybuilders (females = 58.30%) with a history of at least 6 months' regular AAS use prior to data collection. Age of participants ranged from 14 to 56 (*M* = 26.89, *SD* = 7.12) years. Other participant characteristics are presented in Table 1.

**Table 1:** Sample characteristics.

**Variable**	**Overall (*****N*** **=** **206)**		**Male (*n* = 86)**	**Female (*n* = 120)**
	**Range**	**Mean (*SD*)**	**Mean (*SD*)**	**Mean (*SD*)**
Age (years)	14–56	26.89 (7.12)	26.69 (6.72)	27.04 (7.44)
Height (cm)	154–192	171.54 (9.66)	180.46 (6.09)	165.11 (5.91)
Weight (kg)	45–170	71.53 (17.23)	84.64 (16.23)	62.06 (10.41)
**Weight training**				
Total years	1–36	3.28 (4.02)	3.52 (4.96)	3.08 (3.04)
Days per week	1–7	3.40 (1.49)	3.97 (1.62)	2.97 (1.23)
Hours per week	1–20	5.12 (4.66)	6.19 (5.85)	4.37 (3.38)
AAS use duration (years)	1–3	1.33 (0.56)	1.51 (0.66)	1.19 (0.43)
		***N*** **(%)**	***n*** **(%)**	***n*** **(%)**
**Highest education**				
High School		12 (5.80)	5 (5.80)	7 (5.80)
Diploma		35 (17.00)	16 (18.60)	19 (15.80)
Technician		20 (9.7)	8 (9.30)	12 (10.00)
Bachelor		92 (44.70)	39 (45.30)	53 (44.20)
Master		34 (16.5)	14 (16.30)	20 (16.70)
PhD		10 (4.9)	1 (1.2)	9 (7.50)
**Marriage status**				
Single		153 (74.30)	67 (77.90)	86 (71.70)
Married		52 (25.20)	18 (20.90)	34 (28.30)
**Work status**				
Full time		58 (28.20)	30 (34.90)	28 (23.30)
Part time		28 (13.60)	7 (8.10)	21 (17.50)
Self-employed		38 (18.40)	24 (27.90)	14 (11.70)
Unemployed		34 (16.50)	8 (9.3)	26 (21.70)
Student		40 (19.40)	14 (16.30)	26 (21.70)
**Sports involvement**				
Competitive sport		6 (2.90)	4 (4.70)	2 (1.70)
Recreational sport		34 (16.50)	17 (19.80)	17 (14.20)
Competitive bodybuilding		30 (14.60)	10 (11.60)	20 (16.70)
Recreational bodybuilding		105 (51.00)	52 (60.50)	53 (44.20)
		***N***	***n***	***n***
**AAS type used^a^**				
Anadrol (Oxymetholone)		62	39	23
Anavar (Oxandrolone)		112	57	55
Deca-Durabolin (Nandrolone decanoate)		47	30	17
Depo-Testosterone		7	6	1
Dianabol (Methandrostenolone)		71	38	33
Durabolin		7	2	5
Equipoise (Boldenone)		12	8	4
Finajet (Trenbolone)		12	9	3
Maxibolin (Ethylestrenol)		2	2	0
Methyltestosterone		4	4	0
Primobolan		9	4	5
Stanozolol (Winstrol)		136	96	40
Steroid cocktail		3	1	2
Sustanon		44	21	23
Testosterone		114	66	48

a *Percentages may not add up to 100% due to missing data on the respective items*.

### Measures

#### Demographics

The questionnaire assessed demographic factors including age, sex, weight, height, educational level, marriage, and job status.

#### Sports and Weight Training

We assessed participants' main involvement in sports and exercise as well as their total number of years of regular weight training. We also asked participants how many times per week (number of days and hours) they trained with weights.

#### AAS Use

Current AAS use was assessed with the question “Do you currently use anabolic-androgenic steroids?” (yes/no). In verifying AAS use, participants specified the AAS they used (see Table 1). For the assessment of AAS use duration, participants also indicated how long (years) they had been using AAS regularly.

#### Aggression

We used the Short-Form Buss-Perry Aggression Questionnaire [BPAQ-SF; (43, 44)] to assess aggression. Four items represent physical aggression (e.g., “I have trouble controlling my temper”), three items assess verbal aggression (e.g., “I often find myself disagreeing with people”), three items reflect hostility (e.g., “Other people always seem to get the breaks”), and two items measure anger (e.g., “Sometimes I fly off the handle for no good reason”). Items are answered on a 5-point Likert-type scale ranging from 1 (*Very unlike me*) to 5 (*Very like me*). In the present study, Cronbach's α for physical aggression, verbal aggression, hostility, and anger were 0.73, 0.70, 0.77, and 0.69, respectively.

#### Psychological Distress

We assessed symptoms of psychological distress (anxiety and depression) using the Hospital Anxiety and Depression Scale [HADS; (45, 46)]. HADS contains seven items reflecting anxiety symptoms (HADS-A; e.g., “I feel restless as I have to be on the move”) and seven items reflecting depressive symptoms (HADS-D; e.g., “I have lost interest in my appearance”) experienced over the last week. Items are scored on a 4-point scale (0–3). We computed a composite score of anxiety and depression to indicate psychological distress in general, adhering to previous studies that have shown superior factor loadings of one general factor (47, 48). Scores from HADS utilizing the general factor range from 0 to 56. In the present study, Cronbach's α for psychological distress was 0.82.

### Procedure

Eligibility criteria for participation were being a current: (1) weightlifter and (2) regular AAS user for at least 6 months. Participants were recruited from training centers and sports supplement stores in Tehran, Iran. Data was collected via personal meetings at the training centers and supplement stores. All participants provided oral consent, participation was voluntary and anonymous, and participants could withdraw from participation. Upon participants' consent, a trained research assistant handed paper questionnaires to participants for completion. Clarification about the questionnaire was provided when necessary. No incentive or compensation was provided for participation. Data was collected in June 2019. The study was conducted in accordance with the Declaration of Helsinki. Ethical approval was obtained from Alzahra University in Tehran, Iran.

### Statistical Analysis

Characteristics of the sample was determined in terms of descriptive statistics as means and standard deviations for interval and ratio level variables and by percentages for nominal level variables. The major goal of the current study was to examine patterns of aggression and psychological distress in male and female AAS users, and to investigate whether the identified patterns can be applied to both sexes. First, we conducted a Latent Class Analysis [LCA; (49, 50)] to examine discrete patterns of direct aggression (i.e., physical and verbal aggression), indirect aggression (i.e., anger and hostility), and psychological distress for the entire sample. In the context of present study, the aforementioned indicators were used to estimate latent classes of current AAS users. The Bayesian Information Criterion [BIC; (51)] is the most commonly used fit criteria to select the optimal number of latent classes (50, 52). Models with lower values of information criteria have a better fit to the data (53). A common approach is therefore to select the model with a number of latent classes which shows the lowest BIC (54, 55). The likelihood-ratio chi-square (*L*^2^) is a descriptive measure for assessing model fit and when *L*^2^ is significant it shows that the model does not fit the data. Entropy *R*^2^ and total proportion of adjacent classification error were used as indicators of the quality of the classification.

**Figure 1:**
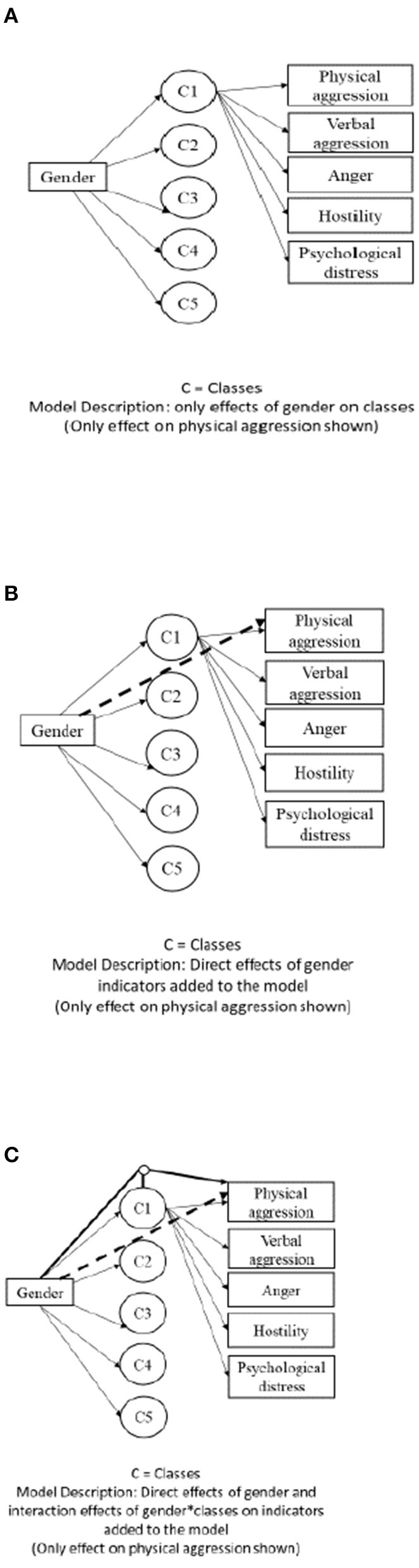
Three types of multiple group latent class models. **(A)** Homogeneous model, **(B)** partially homogeneous model, and **(C)** heterogeneous model.

Measurement invariance is required to determine whether the identified latent classes can be applied to both sexes (53). Thus, after selecting a latent class model for the entire sample, we tested for measurement invariance across sex in order to make a valid comparison across subgroups of male and female AAS users (55, 56). When comparing latent classes across groups, different levels of homogeneity (i.e., measurement invariance) can occur, each of which involves restricting specific sets of model parameters to be equivalent in all groups. There are three prototypical models that differ in the levels of measurement invariance (56). First is the homogeneous model which assumes that the latent classes have the same structure in each group. In this case effects of the group variable on the indicator variables are completely mediated by the latent classes. Hence, the homogeneous model imposes restrictions on the measurement model without deteriorating the fit with the data (Figure 1A).

Second is the partially homogeneous model where some of the model parameters are restricted to be equal in all groups. For example, when differences in answers of participants with different sexes are not associated with differences in a latent class, direct effects occur. Here, the goal is to examine whether the group variable has a direct effect on the indicator variables (Figure 1B). In addition, the heterogeneous model assumes that the latent classes have different structures in each group. In this unrestrictive model, the group variable has three sets of effects including effect on latent classes, direct effect on indicator variables, and interaction effects with latent classes on indicators. Hence, a heterogeneous model lacks any comparability of results across groups as all measurement model parameters are group-specific (Figure 1C).

To test measurement invariance, we conducted homogeneous, partially homogeneous, and heterogeneous multigroup latent class analysis (MLCA) with sex as the grouping variable. Moreover, to relax the assumption of measurement invariance, we assumed that some, but not all, indicators are invariant across sex. We added the direct effect of the grouping variable on each indicator (in the partially homogeneous model). The goal of this step is to check the invariance of each indicator. See Figure 1 for details. A MLCA approach is flexible in the sense that not all latent classes need to be equivalent in order to validly compare results across groups.

Next, for comparing and choosing the best model for the data, we followed a model comparison procedure where difference in likelihood-ratio chi-square (*L*^2^) with a difference in degrees of freedom is used to determine which model is most appropriate. We calculated the difference in *L*^2^ and in degrees of freedom between consecutive nested models. The unrestricted heterogeneous model served as the baseline for this comparison. When moving from the homogenous model to the unrestricted heterogeneous model, if the difference in *L*^2^ between two consecutive nested models (i.e., one model is obtained from a general model by imposing complex and decreasing homogeneity on the former) is significant, the model with more heterogeneity and less degrees of freedom fits the data best (57). Finally, class proportions for each group were examined. Here, participants were assigned to latent classes based on the likelihood of belonging to a class using the proportional assignment rule (57, 58). We used the Walt test to examine the relationship between sex and class membership. Descriptive analyses were conducted using RStudio version 1.1.463 (RStudio, Inc.). The MLCA was performed using advanced LatentGOLD 5.1 (59).

## Results

### Sample Description

The average height (cm) and weight (kg) of female users were 165.11 (*SD* = 5.91) and 62.06 (*SD* = 10.41), respectively. Male AAS users' average height (cm) and weight (kg) were 180.46 (*SD* = 6.09) and 84.64 (*SD* = 16.23), respectively. Female and male AAS users trained with weights, on average, 4.37 (*SD* = 3.38) and 6.19 (*SD* = 5.85) hours per week at the gym. The average AAS use duration was 1.51 (*SD* = 0.66) years for males and 1.19 (*SD* = 0.43) years for females. Oxandrolone (Anavar) (males = 57, females = 55), testosterone (males = 66, females = 48), stanozolol (Winstrol) (males = 96, females = 40), Dianabol (methandrostenolone) (males = 96, females = 33), and Anadrol (oxymetholone) (males = 39, females = 23) were the most used types of AAS among both sexes. See Table 1 for other participant characteristics.

### Patterns of Latent Classes and Measurement Invariance

Fit statistics for the latent classes are presented in Table 2. A 5-class solution was the best model as evident by the lowest BIC (4326.81). The squared log-likelihood (*L*^2^) was 2220.11 (*p* < 0.001), the total proportion of adjacent classification errors was 0.02, and entropy *R*^2^ was 0.94.

**Table 2:** Fit statistics for the latent class analysis.

**Model**	**BIC (LL)**	***N* par**	***L*^**2**^**	**ACE (%)**
1-class	4857.99	75	2878.33	0.00
2-class	4438.13	81	2426.71	0.01
3-class	4349.63	87	2306.45	0.02
4-class	4338.65	93	2263.71	0.03
5-class	4326.81	99	2220.11	0.02
6-class	4330.37	105	2191.91	0.03

*BIC, Bayesian Information Criteria; N par, number of parameters in the model; L^2^, squared log-likelihood; ACE, Adjacent Classification Error*.

As the next step, considering the 5-class solution from the initial Latent Class Analysis (LCA), we conducted a MLCA to assess measurement invariance. Table 3 presents results of the consecutive nested models. When comparing these nested models, we found that sequentially adding direct effects of sex on classes and the interaction between sex and classes did not lead to a significant improvement of fit. Indeed, the Δ*L*^2^ indicated a deterioration of fit. Hence, the model comparison procedure indicated that the homogeneous model fit was better than that of all other models. Hence, sex did not affect measurement variance. From Table 3, we can thus conclude that patterns of aggression and psychological distress are not dependent on the sex of AAS users.

**Table 3:** Fit statistics of the estimated five-class multigroup latent class models.

**Model**	**BIC (LL)**	***L*^**2**^**	***df***	**Comparison**	**Δ*L*^**2**^**	***Sig. result***
Homogeneous model (HM)	4326.81	2220.11	100	—	—	—
**Adding direct effect of sex on one indicator at a time**						
Physical aggression (PA)	4408.85	2500.22	88	HM vs. PA	−148.40	Deterioration
Verbal aggression (VA)	4417.60	2445.45	76	HM vs. VA	−145.99	Deterioration
Anger (A)	4412.08	2397.58	68	HM vs. A	−144.70	Deterioration
Hostility (H)	4369.70	2291.68	56	HM vs. H	−127.40	Deterioration
Partially homogeneous model (PHM) with all direct effects included	4411.33	2190.40	30	HM vs. PHM	29.71	Deterioration
Heterogeneous model (HeM)	2008.66	2315.42	75	HM vs. HeM	−90.65	Deterioration

*BIC, Bayesian Information Criteria; N par, number of parameters in the model; L^2^, squared log-likelihood; ΔL^2^, difference in L^2^ between two consecutive nested model*.

**Figure 2:**
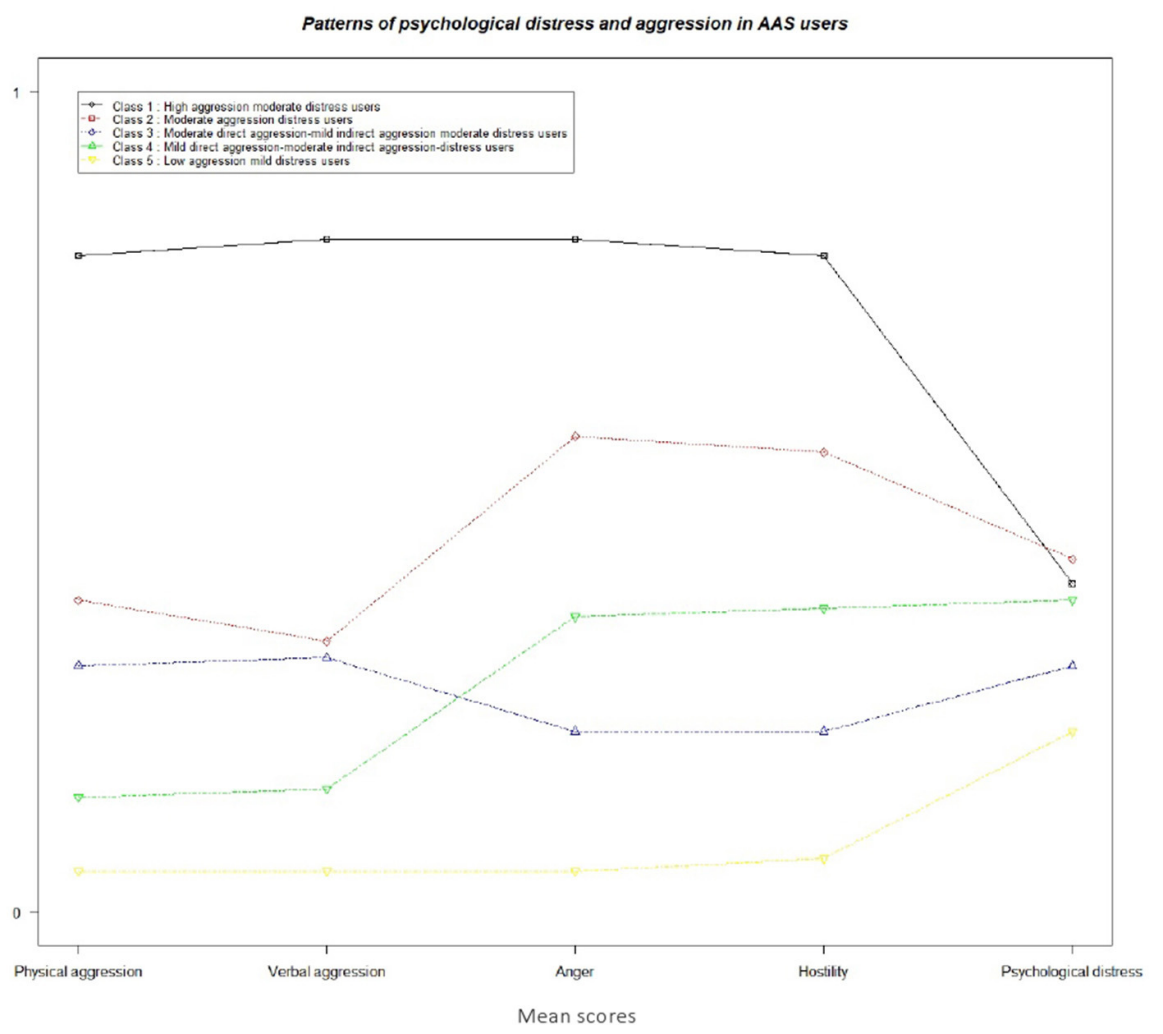
Patterns of aggression and psychological distress in male and female AAS users.

We taxonomized the patterns of the five latent classes on four risk categories: low, mild, moderate, and high. Accordingly, class 1 (high aggression moderate distress users: HAMoD; 7.63%) was characterized by the highest levels of direct (i.e., physical and verbal aggression), indirect (i.e., anger and hostility), and moderate psychological distress. Class 2 (moderate aggression distress users: MoAD; 18.64%) comprised moderate levels of direct and indirect aggression and psychological distress. Class 3 (moderate direct aggression-mild indirect aggression moderate distress users: ModA-MiiA MoD; 11.71%) were characterized by moderate levels of physical and verbal aggression, mild levels of anger and hostility, and moderate psychological distress. Class 4 (mild direct aggression-moderate indirect aggression-distress users: MidA-MoiAD; 11.62%) displayed mild levels of physical and verbal aggression, moderate levels of anger and hostility as well as psychological distress. Class 5 (low aggression mild distress users: LAMiD; 39.06%) were characterized by lowest levels of all the indicators of aggression and mild psychological distress. Figure 2 presents the patterns of aggression and psychological distress for the 5-class model.

In Table 4, we report the class proportions obtained from the selected model. It can be seen that there was an inverse proportional sex distribution of the subgroups. Specifically, the proportion of male AAS users in class 1 (HAMoD) was 91.67% while the proportion of female users was 08.33%. Also, class 2 (MoAD) consisted of 80.42% males and 19.58% females. The third class (ModA-MiiA-MoD) included 32.46% of male AAS users and 67.54% of female AAS users. Class 4 (MidA-MoiAD) consisted of 30.00% of male AAS users and 70.00% of female AAS users. Finally, the proportion of male users in the fifth class (LAMiD) was 10.51% and the proportion of female users was 89.49%. Table 4 indicates results from Wald test. From Table 4, sex was significantly related to class membership (Wald = 31.04, *p* < 0.001) with members of class 1 (HAMoD) more likely to be male (*z* = −5.02, *p* < 0.001) whereas members of class 5 (LAMiD) were more likely to be female (*z* = 3.51, *p* < 0.001).

**Table 4:** Proportions of male and female AAS users in latent classes of aggression and psychological distress.

**Sex**	**HAMoD (%)**	**MoAD (%)**	**ModA-MiiA-MoD (%)**	**MidA-MoiAD (%)**	**LAMiD (%)**
Male	91.67	80.42	32.46	30.00	10.51
Female	8.33	19.58	67.54	70.00	89.49
Total	07.36	18.64	22.95	11.71	39.06
Wald test^§^	−5.02^*^	−0.52	1.19	1.76	3.51^*^

*HAMoD, high aggression moderate distress users (07.63%); MoAD, moderate aggression distress users (18.64%); ModA-MiiA MoD, moderate direct aggression-mild indirect aggression moderate distress users (22.95%); MidA-MioAD, mild direct aggression-moderate indirect aggression-distress users (11.71%); LAMiD, low aggression mild distress users (39.06%)*.

§ *Wald test: 31.04, p < 0.001; female = 1, male = 0*;

* *p < 0.001*.

## Discussion

The present study examined discrete patterns of AAS male and female users based on measures of aggression and psychological distress using a multigroup latent class analysis. We also examined whether the identified patterns are applicable to both sexes. Five latent classes offered the best explanation for the patterns of physical aggression, verbal aggression, anger, hostility, and psychological distress: high aggression moderate distress (7.63%), low aggression mild distress (39.06%), moderate aggression distress (18.64%), moderate direct aggression-mild indirect aggression moderate distress (22.95%), and mild direct aggression-moderate indirect aggression-distress (11.71%). Altogether, the proportional distribution of the identified subgroups indicates that while majority of AAS users are characterized by low to moderate aggression and psychological distress, about 8% display high aggression with mild psychological distress. Moreover, the proportional distribution denotes an idiosyncratic pattern of aggression and psychological distress in the current AAS-using sample, in line with previous indication from a study of APED users (60). The distribution is also in line with findings from previous studies in which AAS use had higher associations with increased hostility, irritability, and anger rather than direct aggression (17, 60). The observed subgroups of AAS users with different profiles of aggression and psychological distress in the current study adds support to a previous systematic review suggesting that aggression and psychological distress are linked to AAS use but not in all subgroups of AAS users (61).

Furthermore, results from the MLCA indicates that a homogenous model is most appropriate when determining male and female AAS users' direct and indirect aggression and psychological distress profiles. Inferably, AAS users' belongingness to specific subgroups is not sex-determined and the patterns of aggression and psychological distress identified in the present study is applicable to both males and females. Relatedly, the inverse proportional sex distribution of the subgroups, with the highly aggressive subgroup having significantly higher proportions of males whereas the low aggression subgroup comprise of significantly higher proportions of females, indicates that a higher proportion of males (compared to females) are at an elevated risk of aggression and psychological distress. This finding can also be explained by evidence of earlier AAS use initiation, more extensive use (e.g., higher weekly doses and higher number of AAS per cycle) as well as a higher prevalence of stacking, polypharmacy and pyramiding in males compared to females (41). Importantly, the present finding of an inverse proportional sex distribution of the subgroups is consistent with evidence from a recent meta-analysis of experimental studies showing that the association between endogenous testosterone levels as well as testosterone administration and aggression are stronger and significant in males but not females (62).

The current study's findings can inform preventive as well as harm reduction and treatment interventions on AAS use, and aggression and psychological distress. Preventive interventions on AAS use incorporating education about the potential for aggressive behavior and psychological distress may be beneficial in reducing motivation and intention for AAS initiation (63–65). Similarly, it is important that harm reduction and treatment interventions for AAS use is extended into the amelioration of aggression and psychological distress (64, 66, 67). In this regard, the homogenous gender model identified in this study underlines the indispensability of female AAS users in such policy making particularly in relation to the tendency for aggression and psychological distress. Furthermore, there is a need for more refined approaches to treatment and harm reduction in which the importance of AAS users' different profiles of aggression and psychological distress are taken into consideration.

The present findings should be interpreted in the context of limitations such as our reliance on self-reports which can be problematic for assessing substance use (68), and our inability to draw causal inferences due to the cross-sectional survey design. In the current study the average length of AAS use was 1.33 (*SD* = 0.56) years which equals, at least, two cycles of AAS use (28). However, we do not know whether the participants were “on cycle” or “off cycle” when participating in the data collection. Moreover, the most popular AAS used by both sexes were oxandrolone/Anavar, testosterone, stanozolol/Winstrol, Dianabol/methandrostenolone, and Anadrol/oxymetholone in line with evidence from previous studies (37, 69). From these most frequently used types of AAS, only Anadrol (oxymetholone) is anecdotally associated with increased aggression in humans (70, 71). Hence, it is also plausible that factors such as particular AAS, AAS use method (e.g., “blast and cruise,” “blitz-cycles,” and “stacking”) and polypharmacy (28, 72) as well as personality (73), AAS dependence and cognition (7), and premorbidity such as structural brain abnormalities (6, 74) account for the differences between the subgroups identified in the present study and further studies are encouraged to explore these.

Despite the abovementioned limitations, the current study has some notable strengths. First, to our knowledge, the current study represents a novel approach in examining discrete subgroups of aggression and psychological distress among male and female AAS users in particular. Another strength is our use of MLCA in the identification of subgroups and role of sex as this method is empirically superior to traditional categorization using cut-off points (34). In addition, the preponderance of AAS research so far has been conducted largely on male samples in Western countries (3, 36). Further research using longitudinal designs may elucidate transitions between latent classes. An examination of the psychosocial correlates of AAS users' belongingness to the subgroups identified in the present study may also be interesting. Finally, it could be of interest to replicate these subgroups and investigate the AAS types associated with members of each subgroup.

## Conclusion

Using MLCA, we investigated the patterns of male and female AAS users' aggression and psychological distress and whether the identified patterns can be applied to both sexes. We identified five discrete patterns of direct and indirect aggression and psychological distress with about 8% displaying the highest levels of aggression and mild psychological distress. Our findings also indicate that patterns of aggression and psychological distress are applicable to both male and female AAS users. Here, members of the high aggression and moderate psychological distress subgroup are more likely to be male whereas members of the low, mild, and/or moderate aggression and psychological distress subgroup are more likely to be female. Our study provides pioneering empirical evidence of the unique patterns of aggression and psychological distress among male and female AAS users. Our findings can be useful for preventive, harm reduction and treatment interventions on AAS use, and aggression and psychological distress.

## Data Availability Statement

The raw data supporting the conclusions of this article will be made available by the authors, without undue reservation.

## Ethics Statement

Written informed consent was not obtained from the minor(s)' legal guardian/next of kin for the publication of any potentially identifiable images or data included in this article.

## Author Contributions

RC, DS, and SP designed the study. RC coordinated the data collection and drafted the first version of the manuscript. RC and GN conducted analyses of the data. RC, GN, SP, and DS were involved in data interpretation and revision of the manuscript. All authors approved the final manuscript before submission.

## Conflict of Interest

The authors declare that the research was conducted in the absence of any commercial or financial relationships that could be construed as a potential conflict of interest.

## References

[1] Sagoe D Molde H Andreassen CS Torsheim T Pallesen S. The global epidemiology of anabolic-androgenic steroid use: a meta-analysis and meta-regression analysis. Ann Epidemiol. (2014) 24:383–98. 10.1016/j.annepidem.2014.01.009 24582699

[2] Cheung AS Grossmann M. Physiological basis behind ergogenic effects of anabolic androgens. Mol Cell Endocrinol. (2018) 464:14–20. 10.1016/j.mce.2017.01.047 28159654

[3] Sagoe D Andreassen CS Pallesen S. The aetiology and trajectory of anabolic-androgenic steroid use initiation: a systematic review and synthesis of qualitative research. Subst Abuse Treat Prevent Policy. (2014) 9:27–40. 10.1186/1747-597X-9-27 24984881 PMC4091955

[4] Bensoussan Y Anderson J. Case report: the long-term effects of anabolic steroids on the female voice over a 20-year period. Clin Case Rep. (2019) 7:1067. 10.1002/ccr3.2084 31110747 PMC6509898

[5] Cunningham RL Lumia AR McGinnis MY. Androgenic anabolic steroid exposure during adolescence: ramifications for brain development and behavior. Hormones Behav. (2013) 64:350–6. 10.1016/j.yhbeh.2012.12.009 23274699 PMC3633688

[6] Hauger LE Westlye LT Fjell AM Walhovd KB Bjørnebekk A. Structural brain characteristics of anabolic androgenic steroid dependence in men. Addiction. (2019) 114:1405–15. 10.1111/add.14629 30955206 PMC6767448

[7] Hauger LE Sagoe D Vaskinn A Arnevik EA Leknes S Jørstad ML. Anabolic androgenic steroid dependence is associated with impaired emotion recognition. Psychopharmacology. (2019) 236:2667–76. 10.1007/s00213-019-05239-7 30941469

[8] Kaufman MJ Kanayama G Hudson JI Pope HG. Supraphysiologic-dose anabolic-androgenic steroid use: a risk factor for dementia? Neurosci Biobehav Rev. (2019) 100:180–207. 10.1016/j.neubiorev.2019.02.014 30817935 PMC6451684

[9] Pope H Katz D. Bodybuilder's psychosis. Lancet. (1987) 329:863. 10.1016/S0140-6736(87)91642-4 2882266

[10] Tragger J. Beware “roid rage” in athletes. Med Tribune. (1988) 29:1–13.

[11] Lundholm L Käll K Wallin S Thiblin I. Use of anabolic androgenic steroids in substance abusers arrested for crime. Drug Alcoh Depend. (2010) 111:222–6. 10.1016/j.drugalcdep.2010.04.020 20627426

[12] Chegeni R Pallesen S McVeigh J Sagoe D. Anabolic-androgenic steroid administration increases self-reported aggression in healthy males: a systematic review and meta-analysis of experimental studies. Psychopharmacology. (2021) 10.1007/s00213-021-05818-7 33745011 PMC8233285

[13] Sagoe D Mentzoni RA Hanss D Pallesen S. Aggression is associated with increased anabolic-androgenic steroid use contemplation among adolescents. Subst Use Misuse. (2016) 51:1462–9. 10.1080/10826084.2016.1186696 27356242

[14] Jenssen IH Johannessen KB. Aggression and body image concerns among anabolic androgenic steroid users, contemplators, and controls in Norway. Body Image. (2015) 12:6–13. 10.1016/j.bodyim.2014.08.009 25261635

[15] Perry PJ Lund BC Deninger MJ Kutscher EC Schneider J. Anabolic steroid use in weightlifters and bodybuilders: an internet survey of drug utilization. Clinical J Sport Med. (2005) 15:326–30. 10.1097/01.jsm.0000180872.22426.bb 16162991

[16] Værøy H. Aggression questionnaire scores in extremely violent male prisoners, male bodybuilders, and healthy non-violent men. Open J Psychiatry. (2013) 3:293. 10.4236/ojpsych.2013.33029

[17] Panagiotidis D Clemens B Habel U Schneider F Schneider I Wagels L. Exogenous testosterone in a non-social provocation paradigm potentiates anger but not behavioral aggression. Eur Neuropsychopharmacol. (2017) 27:1172–84. 10.1016/j.euroneuro.2017.07.006 28939164

[18] Pope HG Kouri EM Hudson JI. Effects of supraphysiologic doses of testosterone on mood and aggression in normal men: a randomized controlled trial. Archiv Gen Psychiatry. (2000) 57:133–40. 10.1001/archpsyc.57.2.133 10665615

[19] Wagels L Votinov M Kellermann T Eisert A Beyer C Habel U. Exogenous testosterone enhances the reactivity to social provocation in males. Front Behav Neurosci. (2018) 12:37. 10.3389/fnbeh.2018.00037 29551966 PMC5840258

[20] Carré JM Geniole SN Ortiz TL Bird BM Videto A Bonin PL. Exogenous testosterone rapidly increases aggressive behavior in dominant and impulsive men. Biol Psychiatry. (2017) 82:249–56. 10.1016/j.biopsych.2016.06.009 27524498

[21] Chegeni R Sagoe D Mentzoni RA Pallesen S. Aggression and anabolic–androgenic steroid use intent in adolescents: a longitudinal study. Subst Use Misuse. (2019) 54:1509–18. 10.1080/10826084.2019.1589522 30931688

[22] O'Connor DB Archer J Hair WM Wu FC. Exogenous testosterone, aggression, and mood in eugonadal and hypogonadal men. Physiol Behav. (2002) 75:557–66. 10.1016/S0031-9384(02)00647-9 12062320

[23] Babor TF Caetano R. Subtypes of substance dependence and abuse: implications for diagnostic classification and empirical research. Addiction. (2006) 101:104–10. 10.1111/j.1360-0443.2006.01595.x 16930166

[24] Lanza ST Rhoades BL. Latent class analysis: an alternative perspective on subgroup analysis in prevention and treatment. Prevent Sci. (2013) 14:157–68. 10.1007/s11121-011-0201-1 21318625 PMC3173585

[25] Evans-Polce R Lanza S Maggs J. Heterogeneity of alcohol, tobacco, and other substance use behaviors in US college students: a latent class analysis. Addict Behav. (2016) 53:80–5. 10.1016/j.addbeh.2015.10.010 26476004 PMC4712642

[26] Morean ME Kong G Camenga DR Cavallo DA Simon P Krishnan-Sarin S. Latent class analysis of current e-cigarette and other substance use in high school students. Drug Alcoh Depend. (2016) 161:292–7. 10.1016/j.drugalcdep.2016.02.018 26922282 PMC4809435

[27] Christiansen AV Vinther AS Liokaftos D. Outline of a typology of men's use of anabolic androgenic steroids in fitness and strength training environments. Drugs Educ Prevent Policy. (2017) 24:295–305. 10.1080/09687637.2016.1231173

[28] Hildebrandt T Langenbucher JW Carr SJ Sanjuan P. Modeling population heterogeneity in appearance-and performance-enhancing drug (APED) use: applications of mixture modeling in 400 regular APED users. J Abnor Psychol. (2007) 116:717–33. 10.1037/0021-843X.116.4.717 18020718

[29] Sagoe D. Nonmedical anabolic-androgenic steroid use: Prevalence, attitudes, and social perception (PhD thesis). University of Bergen, Bergen, Norway (2014).

[30] Zahnow R McVeigh J Bates G Hope V Kean J Campbell J. Identifying a typology of men who use anabolic androgenic steroids (AAS). Int J Drug Policy. (2018) 55:105–12. 10.1016/j.drugpo.2018.02.022 29525360

[31] Havnes IA Jørstad ML Wisløff C. Anabolic-androgenic steroid users receiving health-related information; health problems, motivations to quit and treatment desires. Subst Abuse Treat Prevent Policy. (2019) 14:20–31. 10.1186/s13011-019-0206-5 31096999 PMC6524231

[32] Pagonis P Angelopoulos N Koukoulis GN Hadjichristodoulou CS. Psychiatric side effects induced by supraphysiological doses of combinations of anabolic steroids correlate to the severity of abuse. Eur Psychiatry. (2006) 21:551–62. 10.1016/j.eurpsy.2005.09.001 16356691

[33] Lazarsfeld PF Henry NW. Latent Structure Analysis. Boston, MA: Houghton Mifflin (1968).

[34] Berzofsky M Biemer P Kalsbeek W. A brief history of classification error models. Section on Survey Research Methods-JSM. (2008). 3667–73.

[35] Kankaraš M Vermunt JK. Simultaneous latent class analysis across groups. In A. C. Michalos, editors. Encyclopedia of Quality of Life and Well-Being Research. Dordrecht: Springer (2014) p. 5969–74.

[36] Sagoe D Pallesen S. Androgen abuse epidemiology. Curr Opin Endocrinol Diabetes Obes. (2018) 25:185–94. 10.1097/MED.0000000000000403 29369917

[37] Abrahin O Félix Souza NS de Sousa EC Santos AM Bahrke MS. Anabolic–androgenic steroid use among Brazilian women: an exploratory investigation. J Subst Use. (2017) 22:246–52. 10.1080/14659891.2016.1179806

[38] Börjesson A Gårevik N Dahl ML Rane A Ekström L. Recruitment to doping and help-seeking behavior of eight female AAS users. Subst Abuse Treat Prevent Policy. (2016) 11:11. 10.1186/s13011-016-0056-3 26945991 PMC4779574

[39] Grogan S Shepherd S Evans R Wright S Hunter G. Experiences of anabolic steroid use: in-depth interviews with men and women body builders. J Health Psychol. (2006) 11:845–56. 10.1177/1359105306069080 17035257

[40] Havnes IA Jørstad ML Innerdal I Bjørnebekk A. Anabolic-androgenic steroid use among women–A qualitative study on experiences of masculinizing, gonadal and sexual effects. Int J Drug Policy. (2020) 102876. 10.1016/j.drugpo.2020.102876 32736958

[41] Ip EJ Barnett MJ Tenerowicz MJ Kim JA Wei H Perry PJ. Women and anabolic steroids: An analysis of a dozen users. Clin J Sport Med. (2010) 20:475–81. 10.1097/JSM.0b013e3181fb5370 21079445

[42] Santos AM. Body Image and Bodybuilding Culture in Three Brazilian Gyms: A Qualitative Analysis. Salvador: Federal University of Bahia, Salvador, Brazil (2014).

[43] Diamond PM Magaletta PR. The short-form Buss–Perry aggression questionnaire (BPAQ-SF). A validation study with federal offenders. Assessment. (2006) 13:227–40. 10.1177/1073191106287666 16880276

[44] Samani S. Study of reliability and validity of the Buss and Perry's aggression questionnaire. Iranian J Psychiatry Clin Psychol. (2008) 13:359–65.

[45] Montazeri A Vahdaninia M Ebrahimi M Jarvandi S. The Hospital Anxiety and Depression Scale (HADS): translation and validation study of the Iranian version. Health Qual Life Outcome. (2003) 1:14–8. 10.1186/1477-7525-1-14 12816545 PMC161819

[46] Zigmond AS Snaith RP. The hospital anxiety and depression scale. Acta Psychiatr Scand. (1983) 67:361–70. 10.1111/j.1600-0447.1983.tb09716.x 6880820

[47] Bjelland I Dahl AA Haug TT Neckelmann D. The validity of the Hospital Anxiety and Depression Scale: an updated literature review. J Psychosomatic Res. (2002) 52:69–77. 10.1016/S0022-3999(01)00296-3 11832252

[48] Kjærgaard M Arfwedson Wang CE Waterloo K Jorde R. A study of the psychometric properties of the Beck Depression Inventory-II, the Montgomery and Åsberg Depression Rating Scale, and the Hospital Anxiety and Depression Scale in a sample from a healthy population. Scand J Psychol. (2014) 55:83–9. 10.1111/sjop.12090 24256317

[49] Bakk Z Vermunt JK. Robustness of stepwise latent class modeling with continuous distal outcomes. Struct Equ Model. (2016) 23:20–31. 10.1080/10705511.2014.95510431619843

[50] Collins LM Lanza ST. Latent class and latent transition analysis: with applications in the social, behavioral, and health sciences. New York, NY: John Wiley and Sons (2010).

[51] Schwarz G. Estimating the dimension of a model. Ann Stat. (1978) 6:461–4.

[52] Nylund KL Asparouhov T Muthén BO. Deciding on the number of classes in latent class analysis and growth mixture modeling: a Monte Carlo simulation study. Struct Equ Model Multidisc J. (2007) 14:535–69. 10.1080/10705510701575396

[53] Kankaraš M Moors G Vermunt JK. Testing for measurement invariance with latent class analysis. In Davidov E, Schmidt P, Billiet J, editors. Cross-Cultural Analysis: Methods and Applications. New York, NY: Routledge (2010). p. 359–84.

[54] Hagenaars JA. Categorical Longitudinal Data Log-Linear Analysis of Panel, Trend and Cohort Data. Newbury Park, CA: Sage Publications Inc. (1990).

[55] McCutcheon AL. Latent Class Analysis (No. 64). Beverly Hills, CA: Sage (1987).

[56] Clogg CC Goodman LA. Simultaneous latent structure analysis in several groups. Sociol Methodol. (1985) 15:81–110. 10.2307/270847

[57] Magidson J Vermunt JK. Latent class modelling as a probabilistic extension of K-means clustering. Quirk's Market Res Rev. (2002) 3:77–80.

[58] Goodman LA. On the assignment of individuals to latent classes. Sociol Methodol. (2007) 37:1–22. 10.1111/j.1467-9531.2007.00184.x

[59] Vermunt JK Magidson J. Technical Guide for Latent GOLD 5.1: Basic, Advanced, and Syntax. Belmont, MA: Statistical Innovations Inc. (2016).

[60] Hildebrandt T Langenbucher JW Flores A Harty S Berlin HA. The influence of age of onset and acute anabolic steroid exposure on cognitive performance, impulsivity, and aggression in men. Psychol Addict Behav. (2014) 28:1096–104. 10.1037/a0036482 24841181 PMC4237679

[61] Trenton AJ Currier GW. Behavioural manifestations of anabolic steroid use. CNS Drugs. (2005) 19:571–95. 10.2165/00023210-200519070-00002 15984895

[62] Geniole SN Bird BM McVittie JS Purcell RB Archer J Carré JM. Is testosterone linked to human aggression? A meta-analytic examination of the relationship between baseline, dynamic, and manipulated testosterone on human aggression. Hormones Behav. (2019) 123:104644. 10.1016/j.yhbeh.2019.104644 31785281

[63] Sagoe D Holden G Rise ENK Torgersen T Paulsen G Krosshaug T. Doping prevention through anti-doping education and practical strength training: the Hercules program. Perform Enhanc Health. (2016) 5:24–30. 10.1016/j.peh.2016.01.001

[64] Bates G McVeigh J Leavey C. Looking beyond the provision of injecting equipment to people who use anabolic androgenic steroids: harm reduction and behavior change goals for UK policy. Contemp Drug Prob. (2021). 10.1177/0091450921998701

[65] Petróczi A Dodge T Backhouse SH Adesanwo C. Review of the literature on negative health risks based interventions to guide anabolic steroid misuse prevention. Perform Enhanc Health. (2014) 3:31–44. 10.1016/j.peh.2014.08.001

[66] Bates G Van Hout MC Teck JTW McVeigh J. Treatments for people who use anabolic androgenic steroids: a scoping review. Harm Reduct J. (2019) 16:1–15. 10.1186/s12954-019-0343-1 31888665 PMC6937954

[67] Pope H. G. Jr. Kanayama G. Neurobiology and treatment of anabolic-androgenic steroid-related disorders. In: Brady KT, Levin FR, Galanter MC, Kleber DH, editos. The American Psychiatric Association Publishing Textbook of Substance Use Disorder Treatment. Washington, DC: American Psychiatric Association (2021). p. 315–31.

[68] Hickman M Taylor C Chatterjee A Degenhardt L Frischer M Hay G. Estimating the prevalence of problematic drug use: a review of methods and their application. Bull Narcot. (2002) 54:15–32. 10.1016/j.drugopo.2005.02.05

[69] Gruber AJ Pope HG Jr. Psychiatric and medical effects of anabolic-androgenic steroid use in women. Psychother Psychosomat. (2000) 69:19–26. 10.1159/000012362 10601831

[70] Barker S. Oxymethalone and aggression. Br J Psychiatry. (1987) 151:564. 10.1192/S0007125000217698 3447685

[71] Llewellyn W. Anabolics. Jupiter, FL: Molecular Nutrition LLC. (2011).

[72] Lundholm L Frisell T Lichtenstein P Langstrom N. Anabolic androgenic steroids and violent offending: confounding by polysubstance abuse among 10365 general population men. Addiction. (2015) 110:100–8. 10.1111/add.12715 25170826

[73] Hauger LE Havnes IA Jørstad ML Bjørnebekk A. Anabolic androgenic steroids, antisocial personality traits, aggression and violence. Drug Alcoh Depend. (2021) 221:108604. 10.1016/j.drugalcdep.2021.108604 33621808

[74] Bjørnebekk A Walhovd KB Jørstad ML Due-Tønnessen P Hullstein IR Fjell AM. Structural brain imaging of long-term anabolic-androgenic steroid users and nonusing weightlifters. Biol Psychiatry. (2017) 82:294–302. 10.1016/j.biopsych.2016.06.017 27616036

